# Studies on High-Temperature Evolution of Low-Loaded Pd Three-Way Catalysts Prepared by Laser Electrodispersion

**DOI:** 10.3390/ma16093501

**Published:** 2023-05-01

**Authors:** Tatiana N. Rostovshchikova, Marina I. Shilina, Sergey A. Gurevich, Denis A. Yavsin, Grigory B. Veselov, Vladimir O. Stoyanovskii, Aleksey A. Vedyagin

**Affiliations:** 1 Department of Chemistry, Lomonosov Moscow State University, 1/3 Leninskie Gory, 119991 Moscow, Russia; 2 Ioffe Physico-Technical Institute, Russian Academy of Sciences, 26 Politechnicheskaya Str., 194021 Saint Petersburg, Russia; 3 Boreskov Institute of Catalysis, 5 Lavrentyev Avenue, 630090 Novosibirsk, Russia

**Keywords:** laser electrodispersion, palladium nanoparticles, alumina support, three-way catalysts, thermal stability, characterization

## Abstract

Pd/Al_2_O_3_ catalyst of the “crust” type with Pd loading of 0.03 wt.% was prepared by the deposition of 2 nm Pd particles on the outer surface of the alumina support using laser electrodispersion (LED). This technique differs from a standard laser ablation into a liquid in that the formation of monodisperse nanoparticles occurs in the laser torch plasma in a vacuum. As is found, the LED-prepared catalyst surpasses Pd-containing three-way catalysts, obtained by conventional chemical synthesis, in activity and stability in CO oxidation under prompt thermal aging conditions. Thus, the LED-prepared Pd/Al_2_O_3_ catalyst showed the best thermal stability up to 1000 °C. The present research is focused on the study of the high-temperature evolution of the Pd/Al_2_O_3_ catalyst in two reaction mixtures by a set of physicochemical methods (transmission electron microscopy, X-ray photoelectron spectroscopy, and diffuse reflectance UV-vis spectroscopy). In order to follow the dispersion of the Pd nanoparticles during the thermal aging procedure, the testing reaction of ethane hydrogenolysis was also applied. The possible reasons for the high stability of LED-prepared catalysts are suggested.

## 1. Introduction

In the past few decades, emissions of automobile exhaust gases became one of the main sources of atmospheric pollution [1,2,3]. Due to the incomplete combustion of fuel in internal combustion engines, exhaust gases contain considerable amounts of carbon monoxide and hydrocarbons [4]. Another important contaminant contained in exhaust gases, nitrogen oxides (NO_x_), originates from the reaction between N_2_ and O_2_ during the combustion of fuel. As is abundantly reported in the literature [5,6,7,8], long-term exposure to all these contaminants has a significantly negative impact on human health. The use of so-called three-way catalysts (TWC) allows for reducing their concentration, thus lowering their impact on the environment significantly [9,10]. TWCs contain in their composition noble metals such as palladium, platinum, and rhodium that play the role of active components [11,12,13,14]. It should be noted that palladium and platinum are active in the oxidation of CO and hydrocarbons, while rhodium is important for the reduction of NO_x_. Therefore, under near-stoichiometric conditions, such catalysts containing Rh and Pd, and/or Pt effectively neutralize all three types of contaminants.

The ever-toughening requirements and emission standards aimed to reduce pollutants contained in the exhaust gases from vehicles force the catalyst manufacturers to increase constantly the concentration of noble metals in order to improve the catalyst activity, which significantly raises the cost of catalytic compositions. Thereby, the production of highly active and stable compositions with a reduced content of precious metals looks like a very promising direction for the development of three-way catalysts [15].

On the other hand, prolonged exposure of TWCs to high temperatures under the reaction medium conditions causes another significant problem—their rapid deactivation. Among several reasons for deactivation processes that occur during the TWC operation, thermal instability is the most serious one [16,17,18,19,20,21]. Therefore, commercially produced aluminum oxide, Al_2_O_3_, traditionally used as a support for TWC, possesses appropriate thermal stability. Moreover, the addition of cerium, zirconium, lanthanum, or barium oxides to alumina makes it even more thermally stable [22,23,24]. The chemical deactivation of TWCs was mostly caused by a high content of lead and sulfur in fuel [25,26]. Among the mentioned noble metals, palladium is affected by sulfur more significantly [27]. However, up to now, an increase in fuel quality reflected in lowered sulfur content allowed palladium to become the main component of TWCs [10]. Despite the lower activity of Pd in the reduction of NO_x_ compared to Rh, it demonstrates noticeably higher activity in oxidation reactions [28]. In addition, the high cost and scarcity of Rh are of great concern. Therefore, Pd-only TWCs received considerable attention over the past few decades [29,30,31,32,33,34,35]. Pd-based catalysts demonstrate particularly high efficiency in methane oxidation in a mode of periodic rich/lean operation [36]. CO oxidation is widely used as a model reaction in the fundamental research of heterogeneous catalytic processes, but the evolution of a catalytic system at high temperatures remains poorly understood [37].

In cases when palladium is used as an active component, the main deactivation mechanism is the agglomeration and sintering of metal particles [38,39,40]. As is known, the stability of palladium particles is significantly higher under oxidative (lean) conditions if compared to reductive (rich) ones [41]. This can be explained by the fact that palladium metal is significantly more prone to sintering compared to PdO. Moreover, the redispersion of palladium particles is possible under oxidative conditions, leading to the restoration of its initial activity [41,42,43,44]. The size of palladium particles determines the possibility of the redispersion phenomenon. As was found by Lupescu et al. [42], the formation of PdO species and their subsequent effective redispersion require a size of metallic Pd particles smaller than 8.8 nm. According to the data of differential thermal analysis in an air atmosphere, for Pd/Al_2_O_3_ catalysts, the thermal decomposition of PdO occurs at temperatures of 750–900 °C, and the formation of PdO at the cooling stage takes place within the temperature range of 550–700 °C [45,46]. Therefore, the understanding of the high-temperature evolution of palladium species is highly important to create efficient and thermally stable TWCs.

The metal-support interaction (MSI) is known to play a significant role in stabilizing palladium in the dispersed state [47,48,49,50]. The stabilization of palladium at the donor sites of the Al_2_O_3_ support was confirmed using the spin probe electron paramagnetic resonance spectroscopy method. As previously reported, with an increase in palladium concentration in model Pd/Al_2_O_3_ catalysts, the concentration of anion radicals formed during the adsorption of trinitrobenzene also increases. The maximum concentration of these anion radicals is achieved when the Pd concentration is 0.5 wt.%. This agrees well with the formation of larger PdO particles, the presence of which was confirmed by the temperature-programmed reduction method. The role of the penta-coordinated Al^3+^ sites on the surface of Al_2_O_3_ has been proven by Duan et al. [51]. Such sites can interact with the surface species PdO_x_, thus increasing their stability and activity significantly. It should also be noted that in the case of Pd/Al_2_O_3_ catalysts, core–shell structures can be formed, consisting of metallic palladium core and palladium–aluminate shell. Such structures exhibit excellent stability under CO oxidation conditions [52].

The effects of the particle size and morphology of the active component on the activity of Pd/Al_2_O_3_ catalysts is well known. Haneda et al. compared the catalytic activity in CO and propylene oxidation reactions of the catalysts with particle sizes varied in a range of 4–14 nm [53]. They found that the concentration of PdO forms decreases with increasing particle size, which has a positive effect on the rate of propylene oxidation. Similarly, in the case of methane oxidation over Pd/γ-Al_2_O_3_ catalysts, the activity grows monotonously with an increase in the particle size [54,55]. It is important to note that the crystalline phase of alumina influences the morphology of Pd particles and the strength of their interaction with the support. Thus, in the case of Pd/θ-Al_2_O_3_ and Pd/α-Al_2_O_3_ catalysts, the MSI is weakened, which leads to the formation of well-faceted Pd particles with a higher concentration of step sites, which appear to be more active in the methane oxidation reaction [54]. The same MSI effect was reported for bimetallic Pd-Rh catalyst supported on δ-Al_2_O_3_ [56]. It should be emphasized that MSI plays an especially important role in catalytic activity and thermal stability of Pd-based TWCs, as was revealed by a number of research groups [57,58,59,60,61]. Although the CO oxidation reaction shows structural insensitivity in the particle size range of 4–14 nm [53], studies in an extended particle size range (starting from 1.5 nm) revealed that the greatest specific activity is observed when the particle size is 2 nm [62]. As it was found, both corner sites and (111) planes are more active in CO oxidation in comparison with step sites. At a particle size of 2 nm, the concentration of corner cites is the highest. Interestingly, in the case of Pt/Al_2_O_3_, the optimal particle size also lies in a range of 2–3 nm [63].

Summarizing the aforesaid, the following conclusions can be made. The development of reliable and reproducible methods for the synthesis of TWCs that ensure the production of active metal particles of several nanometers in size is an actual task. Conventional chemical preparation techniques do not provide the required uniformity in coverage of the surface of the support with the nanoparticles of the active component. Moreover, these methods are multistage and involve the use of wet chemistry.

As an alternative, one-step and environmentally friendly methods based on laser ablation are increasingly being used for the deposition of metal particles in the synthesis of catalysts [64,65,66]. Among them, the laser electrodispersion (LED) method developed at the Ioffe Physicotechnical Institute (St. Petersburg, Russia) is an outstanding one [67]. This method allows depositing one-size nanoparticles directly on the surface of the granulated support without the use of a liquid to stabilize them [67,68,69,70,71,72,73,74]. It was shown to be applicable and effective for the synthesis of mono- and bimetallic systems of different compositions: Pd [68,73,74], Pt [69], Ni-Pd [70], Pd-Zn [71], and Pt-Co [72]. Alumina, various zeolites, and carbon materials were used as support in these works. In many catalytic processes, LED-prepared catalysts are superior not only in activity but also in stability if compared to catalysts prepared by conventional chemical methods (incipient wetness impregnation, precipitation from colloidal dispersions, etc.) [67].

Recently, the LED method was applied for the first time to prepare Pd-containing TWCs [74]. It has been shown that the LED-prepared catalyst exhibits superior thermal stability with regard to the reference sample prepared by the incipient wetness impregnation method. The purpose of the present work is to investigate in detail the evolution of the LED-prepared Pd/Al_2_O_3_ catalysts during high-temperature aging procedures. The samples were tested under the prompt thermal aging conditions [24] in a model mixture containing CO, O_2_, NO, and N_2_ only, and in a real mixture additionally containing hydrocarbons. The light-off CO conversion curves were analyzed at the heating and cooling stages. The state of palladium species after aging at various temperatures (800, 900, and 1000 °C) was characterized by a set of physicochemical methods (low-temperature nitrogen adsorption, transmission electron microscopy, X-ray photoelectron spectroscopy, UV-vis spectroscopy, and a testing reaction of ethane hydrogenolysis).

## 2. Materials and Methods

### 2.1. Preparation of the Catalysts

**Figure 1:**
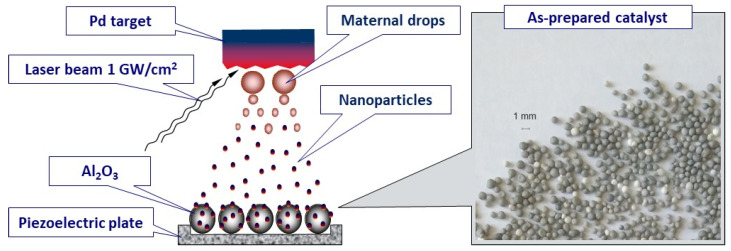
Scheme of preparation of the Pd/A catalyst by laser electrodispersion.

The LED technique [68,75] was used for the deposition of Pd nanoparticles on the surface of γ-Al_2_O_3_ granules 0.4–1.0 mm in size (JSC “Angarsk Plant for Catalysts and Organic Synthesis”, Angarsk, Russia). The scheme of the catalyst preparation process is presented in Figure 1. The process takes place in a vacuum chamber pumped down to 10^−4^ Pa. The Pd target was exposed to radiation from a pulsed YAG:Nd laser (wavelength 1.06 μm, pulse duration 30 ns, pulse energy 120 mJ), which resulted in the generation of a stream of Pd nanoparticles propagating from the target. The details of the particle formation mechanism in the LED process are given elsewhere [67]. A weighted amount of support granules was placed in a special cell equipped with a piezoelectric plate. Due to ultrasonic cell vibration, the support granules were continuously mixed during the deposition. This ensures the uniform coverage of the support surface by Pd nanoparticles. In accordance with the calibration plot obtained previously [75], the time required for the deposition of 150 μg of Pd per 0.5 g of the support was 4 min. According to the atomic absorption spectroscopy (Thermo Fisher Scientific Inc., Waltham, MA, USA) data, the Pd content in the prepared sample was 0.028 wt.%. The catalyst was denoted as Pd/A.

Two reference samples containing 0.2 wt.% Pd were prepared by an incipient wetness impregnation of γ-Al_2_O_3_ with the K_2_[Pd(NO_2_)_4_] solution. The first one was dried at 105 °C for 6 h and then calcined in air at 550 °C for 1 h at a heating rate of 10 °C/min. The second sample, after drying, was calcined in an Ar/H_2_ mixture (5 vol.% of hydrogen) at 300 °C for 1 h at a heating rate of 10 °C/min. In accordance with the previously obtained data, these samples contained dispersed surface Pd^2+^ species and highly dispersed Pd^0^ particles, respectively. They were denoted as Pd^2+^/A-Imp and Pd^0^/A-Imp and used as reference samples in the analysis of UV-vis spectra.

### 2.2. Testing the Catalytic Activity

In order to characterize the catalytic activity, the granules were crushed to obtain a fraction 0.25–0.5 mm in size. A specimen of the fraction (300 mg) was loaded into a quartz reactor with an inner diameter of 5 mm. Two reaction mixtures were used in this study. The first mixture (denoted as a model mixture) consists of 1500 ppm CO, 150 ppm NO, 14 vol.% O_2_, and balance N_2_. The second mixture (denoted as a real mixture) includes 1500 ppm CO, 300 ppm CH_4_, 400 ppm C_3_H_4_, 110 ppm C_6_H_5_CH_3_, 150 ppm NO, 14 vol.% O_2_, and balance N_2_. In all cases, the total flow rate was 334 mL/min. The thermal stability of the samples was examined using a prompt thermal aging (PTA) mode [24]. In the PTA procedure, the sample was tested in 11 temperature-programmed heating–cooling runs. The starting temperature of each run is 50 °C, while the final temperatures rise after each second run and constitute 320 °C (runs #1 and #2), 600 °C (runs #3 and #4), 800 °C (runs #5 and #6), 900 °C (runs #7 and #8), 1000 °C (runs #9 and #10), and 500 °C (run #11). The concentration of CO at the reactor outlet was monitored using a ULTRAMAT 6 gas analyzer (Siemens, Munich, Germany) equipped with an infrared analytical cell. The values of CO conversion (X_CO_) were calculated using Equation (1).

(1) $$X_{\mathrm{CO}}=\frac{\left(C_{0}-C\right)}{C_{0}}\times 100\%$$

where C_0_ is the initial CO concentration (1500 ppm), and C is the current CO concentration at the reactor outlet.

In order to compare the samples, the temperature corresponding to 50% conversion of CO (T_50_) was used as a criterion. The experimental error in the determination of this parameter does not exceed 1 °C. It is worth noting that the classic S-shaped light-off curves were not observed in all the cases. Therefore, differential light-off curves were plotted and analyzed. The maxima on these curves were used for comparison of the samples. To characterize the state of palladium species after various aging temperatures, the catalytic experiments were stopped after the sixth, eighth, and tenth runs, which correspond to aging at 800, 900, and 1000 °C. These samples were labeled as Pd/A-PTA800, Pd/A-PTA900, and Pd/A-PTA1000, respectively.

### 2.3. Characterization of the Materials

Low-temperature nitrogen adsorption/desorption isotherms were recorded using a Sync 200 (3P Instruments GmbH & Co. KG, Odelzhausen, Germany) instrument. The Brunauer–Emmett–Teller (BET) method was used to calculate the values of specific surface area (SSA). The total pore volume (V_total_) was determined from the maximum adsorption value at the highest P/P_0_. The average pore diameter (D_av_) was calculated from the adsorption branch using the Barrett–Joyner–Halenda (BJH) method.

Transmission electron microscopy (TEM) studies were performed using a JEOL JEM 2100F/UHR instrument (Tokyo, Japan) with a resolution of 0.2 nm and a maximum magnification of ×106 times equipped with an energy dispersive X-ray (EDX) accessory. Each sample was prepared for TEM studies as described elsewhere [73,74].

X-ray photoelectron spectra (XPS) were recorded on an Axis Ultra DLD spectrometer (Kratos Analytical, Manchester, U.K.) using monochromatic AlK_α_ radiation with the transmission energy of 160 eV and 40 eV for the survey and high-resolution spectra. The energy scale of the XPS spectra for the samples on Al_2_O_3_ was preliminarily calibrated using an Al2p peak at 74.4 eV as an internal standard. Deconvolution of Pd3d peaks into three components was performed as described previously [73,75]. The following parameters were applied: the Shirley background; GL (30) line shapes; and binding energies of 335.7 eV (Pd^0^), 336.9 eV (PdO), and 338.3 eV (a doublet component associated with Pd(OH)_2_ or Pd^2+^ cations coordinated by the oxygen atoms of the support) [76].

Diffuse reflectance UV-vis spectra were recorded between 190 and 800 nm using a UV-vis spectrometer Varian Cary 300 UV/VIS Bio (Agilent Technologies, Inc., Santa Clara, CA, USA) with Labsphere DRA-CA-3300 integrating sphere (Varian, Inc., Palo Alto, CA, USA) with Spectralon^®^ standard as a reference. The UV–vis spectra were transformed into the Kubelka–Munk function (Equation (2)) [77].

F(R) = *α*/*s* (2)

where *α* is the absorption and *s* is the scattering.

The samples of the support and catalysts were investigated in a naturally hydrated state under atmospheric conditions. Before the study, all samples were ground in an agate mortar until reaching a homogeneous state. The value of energy gap width (*E_g_*) was determined from diffuse reflection spectra using Equation (3) proposed by Tauc, Davis, and Mott.

[F(R)·*hν*]^n^ = *A*(*hν* − *E_g_*) (3)

where *h* is Planck’s constant, *ν* is the radiation frequency, function F(R)~*α* is the absorption coefficient, *E_g_* is the band gap, and *A* is the proportionality factor. Note that *hν* represents the photon energy, and n = 2 represents an allowed direct transition [78].

To characterize the dispersion of the deposited palladium, the samples were examined in a testing reaction of ethane hydrogenolysis. The rate of this reaction correlates with the surface concentration of metal atoms accessible for the reagents [79,80]. The experiments were performed according to the following procedure. A specimen of the fraction (100 mg) was loaded into a quartz reactor. The reactor was fed with a flow of helium mixed with hydrogen, heated to 200 °C, and maintained at such conditions for some time until the system reached a stationary state. After that, ethane was added to the reaction flow for 3 min. Then, a sample of the gas mixture was taken at the reactor outlet and the ethane feed was stopped. Analysis of the outlet gas mixture was carried out using a Crystal-2000M chromatograph (Chromatec Ins., Yoshkar-Ola, Russia) equipped with a flame ionization detector. The He/H_2_ mixture was purged through the sample for 10 min, thus bringing the catalyst’s surface to its initial state. The procedure was repeated 5 times at each temperature point within a range of 200–540 °C.

## 3. Results and Discussion

### 3.1. Catalytic Activity and Thermal Stability of the Samples

**Figure 2:**
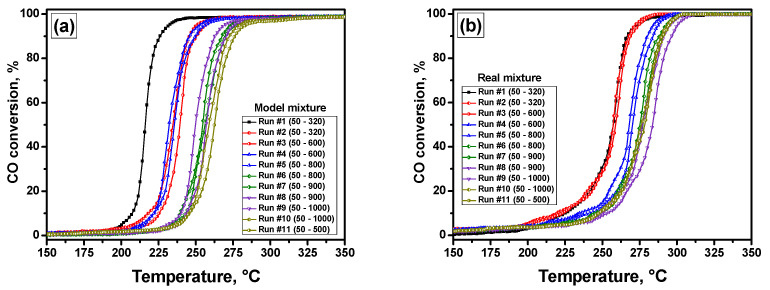
Light-off curves for CO oxidation in the PTA mode over the Pd/A catalyst: (**a**) model mixture; (**b**) real mixture.

The catalytic activity of the LED-prepared Pd/A catalyst was tested in the PTA mode as described above. As is well known, the composition of the reaction mixture noticeably affects both the activity and the stability of the catalyst. Therefore, in the present study, the PTA tests were performed using two reaction mixtures: the model mixture, which consisted of CO, NO, and an excess of O_2_ diluted with N_2_, and the real mixture, which, besides the mentioned components, also contained hydrocarbons, being the common constituents of automobile exhaust gases. Figure 2 presents the light-off curves for the Pd/A sample tested in the PTA mode. All the heating/cooling light-off curves are collected in Figures S2 and S3 (Supplementary Information). The catalyst demonstrates rather high stability in the case of both model (Figure 2a) and real (Figure 2b) mixtures. In the case of a model mixture, the initial activity is noticeably higher. However, the distinctive shift of the second-run curve toward higher temperatures, which illustrates the first step of the catalyst deactivation, is evidently seen. Aging at 800 °C initiates the second deactivation step, after which the catalytic performance seems to be stabilized. In the case of a real mixture, deactivation occurs in three steps after treatment at 600, 800, and 900 °C. Further aging at 1000 °C results in reactivation of the catalyst, which can be associated with the redispersion of Pd species described in the Section 1. Overall, the highest CO conversion values are reached on the Pd/A catalyst at lower temperatures in the absence of hydrocarbons. Although the reaction starts at similar temperatures, the light-off curves are less steep in the case of a real mixture, which corresponds to lower reaction rates. This can be explained by the competitive sorption of hydrocarbons and CO on the surface of Pd nanoparticles. In addition, active oxygen atoms adsorbed on the surface are required for the reaction to take place. Adsorption of hydrocarbons on the surface decreases its coverage with CO and oxygen species, thus shifting the light-off curves toward higher temperatures. It can be supposed that unsaturated hydrocarbons present in the composition of a real mixture, namely toluene and propene, play a decisive role in this process.

**Figure 3:**
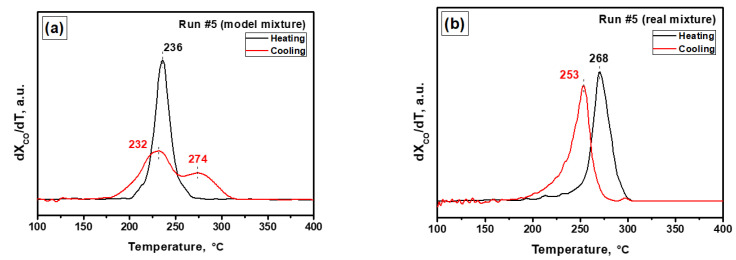
Differential light-off curves for the Pd/A catalyst tested in the PTA mode (fifth run): (**a**) model mixture; (**b**) real mixture.

For a clearer representation of the catalytic results, differential light-off curves were plotted. All the plots are presented in Figures S4 and S5 (Supplementary Information). The positions of maxima for all differential curves are summarized in Table 1. Figure 3a,b demonstrates the differential light-off curves (run #5) for model and real mixtures, respectively. Obviously, an ideal S-shaped curve gives the differential curve with one maximum, the position of which corresponds to T_50_, as is observed for the real mixture (Figure 3b). In the case of the model mixture, the cooling curve shows two maxima at 232 and 274 °C (Figure 3a). Thus, the comparison of maxima positions on the heating and cooling stages for two reaction mixtures can clarify the differences between these two reaction conditions.

In the case of the model mixture, the shape of the heating and cooling curves changes in an interesting manner during the PTA procedure. In the initial state, the curves are S-shaped. Direct temperature hysteresis, when the cooling curve is shifted to the low-temperature region, is observed. Later on, after the first heating to 600 °C (run #3), the cooling curve changes in shape (Figure S2), which is expressed in the appearance of two maxima and a shoulder on the corresponding differential curve (Figure S4). In run #6, a decrease in conversion at the cooling stage begins at temperatures of ~30 °C higher than at the heating stage. As a result of high-temperature aging at 900–1000 °C, the cooling curve is shifted to the lower-temperature region relative to the heating curve. In run #10, a direct hysteresis is observed but the shape of the cooling curve is still different from the S-shaped one. It is worth noting that in run #11, in which the final heating temperature was 500 °C, the maximum is at a substantially higher temperature. Obviously, the shape of the hysteresis loop depends not only on the maximum aging temperature of the catalyst but also on the final heating temperature in the particular catalytic run.

The light-off curves for all heating and cooling runs recorded under the conditions of the real mixture are shown in Figure S3. In this case, a direct temperature hysteresis when the cooling curves were shifted to the low-temperature region by 13–23 °C (Table 1) was observed in all runs. Note that the shape of the curves is close to an ideal S-shaped one. No shift in the light-off curve at the cooling step in run #11 was observed in the case of this mixture.

The presence of temperature hysteresis during CO oxidation over supported precious metals is a complicated phenomenon usually explained by the existence of multiple steady states in the catalytic system, the phase transformations of catalysts, the changes in the surface state of the adsorbed components, and the removal of admixtures from the catalyst surface, which can hinder the reaction [81,82]. Several parameters are known to affect the shape of hysteresis curves: ramping rate, flow rate, the composition of the reaction mixture, and size of metal particles [81]. The exothermicity of the reaction can also play an important role. Another commonly considered reason is the thermal inertia of the catalyst bed. Due to this phenomenon, the cooling curve is usually shifted to lower temperatures when compared to the heating light-off curve (direct hysteresis). In the case of supported palladium, direct hysteresis is often observed [83,84,85,86,87]. However, Lashina et al. reported an inverse hysteresis on palladium foil due to transformations between metallic Pd and bulk PdO taking place during the heating/cooling cycles [82,88]. The appearance of the inverse hysteresis in the case of the Pd/A catalyst can be attributed to a similar phenomenon. Dubbe et al. observed the inverse hysteresis on a diesel oxidation catalyst for CO oxidation in a complex mixture containing H_2_O and CO_2_ [89]. In the case of a real mixture, the presence of hydrocarbons should change the kinetics of the reaction significantly, since the catalyst’s surface is covered with adsorbed hydrocarbons and the products of their partial oxidation. On the other hand, the obtained results highlight the high stability of the Pd/A catalyst under real operation conditions.

Table 2 compares the catalytic activity of the LED-prepared Pd/A catalyst with the activity of previously reported catalysts with higher Pd content. Note that all the samples were tested under similar conditions, thus allowing an adequate comparison. The excellent thermal stability of the LED-prepared catalyst is apparent. For instance, in the case of 0.12 wt.% Pd/Al_2_O_3_ catalyst [15], the T_50_ value in the seventh run (800 °C) was only 3 °C higher, despite the 4-fold higher content of Pd. Another catalyst described in [74] contains a 7-fold higher amount of Pd. It demonstrated catalytic performance similar to the LED-prepared sample after aging at 1000 °C (run #11). In the case of lanthanum oxide-doped catalyst only, the behavior was noticeably better in the first seven runs [90]. However, its stability is not impressive. Therefore, in order to uncover the reasons for the stable performance of the LED-prepared catalyst, a detailed investigation of the Pd state in the Pd/Al_2_O_3_ catalyst was carried out.

### 3.2. Evolution of the Catalyst during the Prompt Thermal Aging Procedure

As known, the textural characteristics of the catalyst affect its catalytic properties. Therefore, the as-prepared and aged samples of the Pd/A catalyst as well as pure alumina support were examined by low-temperature adsorption/desorption. Table 3 presents the results obtained. Whereas the LED technique allows supporting Pd particles on the outer surface of the alumina granules, the textural characteristics of the as-prepared catalyst do not differ significantly from that for pure support. The average pore diameter and the total pore volume remained the same, while the SSA value increased by ~10%.

No noticeable changes in these parameters are observed after the aging at 800 °C. The further increase in aging temperature up to 900 and 1000 °C led to a drop in the SSA and V_total_ values. Along with this, the average pore diameter rises slightly. In general, the used alumina support exhibits appropriate thermal stability. Despite the high values of the aging temperatures, the duration of aging procedures at these temperatures was not long enough. After the temperature-programmed heating to the final temperature, the sample was immediately cooled down. Therefore, the support retains high textural characteristics. As reported previously [15], the SSA value of the alumina-supported catalyst after prolonged aging at 1000 °C was as low as 111 m^2^/g. In terms of the phase composition, the sample was represented by δ-Al_2_O_3_ with an admixture of θ-Al_2_O_3_ and α-Al_2_O_3_ phases. In the case of the Pd/A catalyst, the formation of the δ-Al_2_O_3_ phase during the PTA procedure presumably takes place as well.

**Figure 4:**
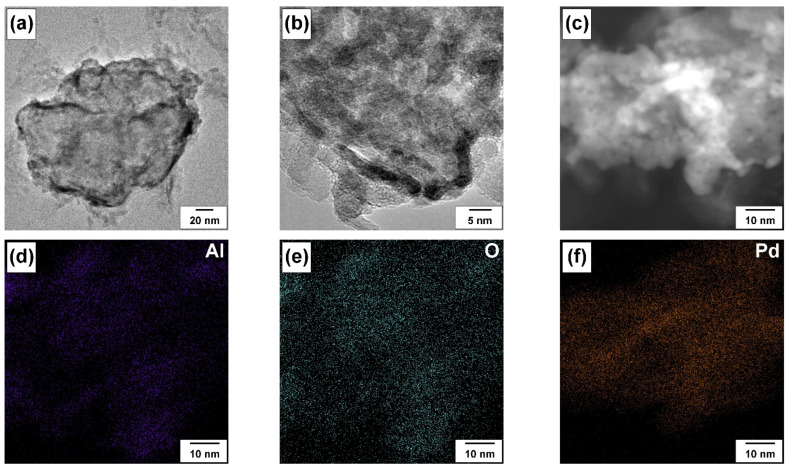
Microscopic data for the as-prepared Pd/A sample: (**a**–**c**) TEM images; (**d**–**f**) elemental mapping.

Then, the as-prepared and PTA-aged samples were investigated by transmission electron microscopy. Typical TEM images of Pd nanoparticles on alumina are shown in Figure 4. In Figure 4a,b, darker areas are clearly visible against a gray background, which, according to the EDX data, can be attributed to palladium-containing particles. Bright Pd-enriched areas are clearly seen in the dark-field image as well (Figure 4c). The particle size cannot be estimated from the obtained images due to the strong overlap of the particles in the images. However, our previous studies, including the Pd deposition directly onto TEM grids, showed that the size of Pd nanoparticles deposited by the LED method is about 2 nm, regardless of the Pd content and the support’s nature [68,71,75].

The element mapping (Figure 4d–f) demonstrates that Pd is uniformly distributed on the surface of alumina. However, the atomic ratios determined by EDX change from 0.1 to 1.7 (Figure S1, Table S1) in the different areas of the Pd/A sample. Still, the observed Pd atomic fraction is very high, considering the low Pd content. A wide spread of values is associated with different thicknesses of the analyzed sample after pretreatment. In addition, as can be seen from Figure 4a,b, the palladium content is significantly higher at the edges of the samples when compared to the inner regions.

**Figure 5:**
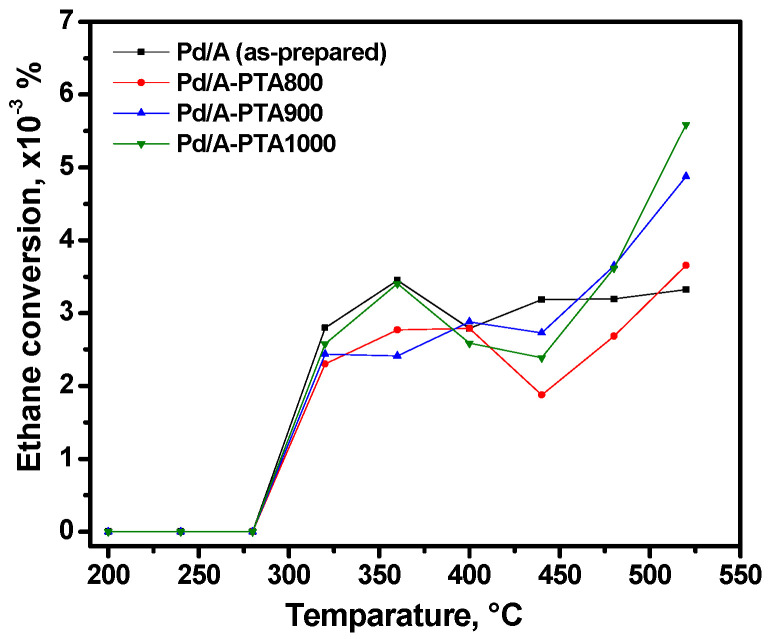
Temperature dependences of ethane conversion for the as-prepared and PTA-aged samples of the Pd/A catalyst.

As previously reported [24], the testing reaction of ethane hydrogenolysis is an informative technique to characterize the dispersion of the supported palladium particles. The rate of this reaction is directly proportional to the metal surface accessible for the reagents. Figure 5 shows the corresponding curves of ethane conversion for the Pd/Al catalyst before and after the catalytic tests. As is seen, the values are very low for all the samples. Such low values are due to the presence of palladium in the form of nanoparticles of uniform shape and size, which are located on the surface of the support very close to each other. On the other hand, the absolute values of ethane conversion are not so important to analyze the performance of the catalysts. Thus, although the reaction starts at the same temperature, the curves differ in shape. In general, it can be supposed that the possible sintering of palladium particles does not lead to significant changes in the value of the accessible surface area of the metal. This correlates well with the results of catalytic tests.

**Figure 6:**
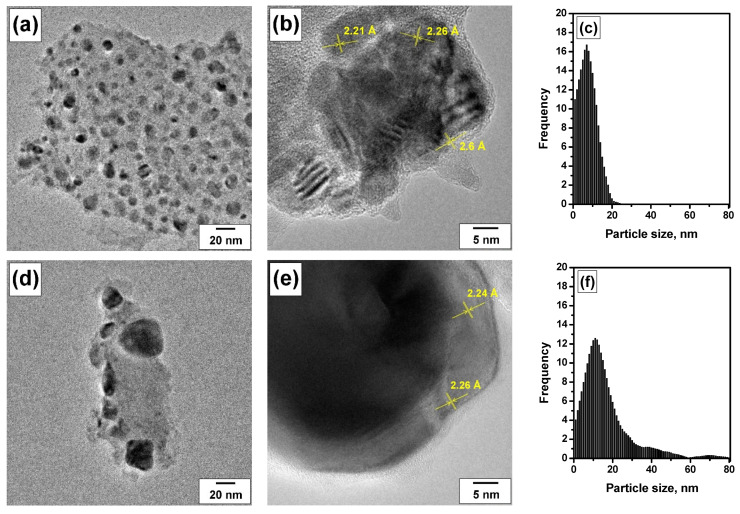

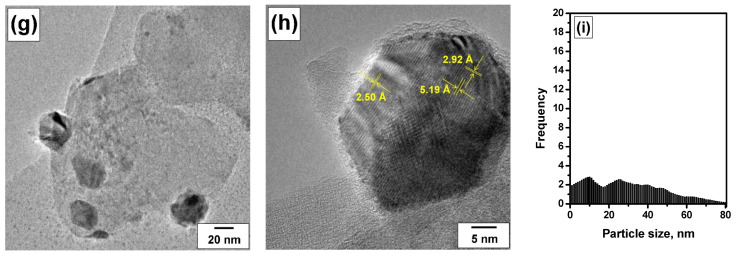
TEM images and corresponding particle size distributions for the Pd/A catalyst tested in the real mixture under the PTA conditions at various temperatures: (**a**–**c**) 800 °C; (**d**–**f**) 900 °C; (**g**–**i**) 1000 °C.

TEM images of the samples tested in the PTA mode at varied temperatures are presented in Figure 6. Unlike the as-prepared sample, in this case, individual Pd/PdO particles are clearly visible, which makes it possible to estimate their size. After 800 °C, significant particle enlargement occurred to an average particle size of about 8 nm (Figure 6a,c). Along with this, the maximum particle size slightly exceeds 20 nm. As is seen from Figure 6b, the particles are close enough to form agglomerates. Analysis of interplanar distances and their comparison with known values for Pd (d_111_ = 2.25 Å) and PdO (d_101_ = 2.65 Å) showed that for the Pd/A-PTA800 sample, the active component is mainly represented by metal Pd particles. However, the formation of PdO was also observed on the surface. PTA treatment at 900 °C initiates further sintering of the particles to an average size of about 17 nm (Figure 6d,f). As a result of sintering, both agglomerates of smaller particles and large particles of metal palladium are formed (Figure 6e). The most significant sintering occurs after aging at 1000 °C. The sintering process of Pd/PdO particles can be clearly seen from the particle size distributions shown in Figure 6i. After this procedure, the average particle size reaches 35 nm. Note that particles of 80 nm and above in size are also observed. In this case, in addition to metal particles, large oxide particles are formed (Figure 6h). The calculated interplanar distances of 2.92 and 5.19 Å are close to the known values of 3.05 and 5.37 Å for perpendicular facets d_100_ and d_001_ in tetragonal PdO. Therefore, TEM studies confirm the high-temperature sintering of Pd particles supposed previously.

**Figure 7:**
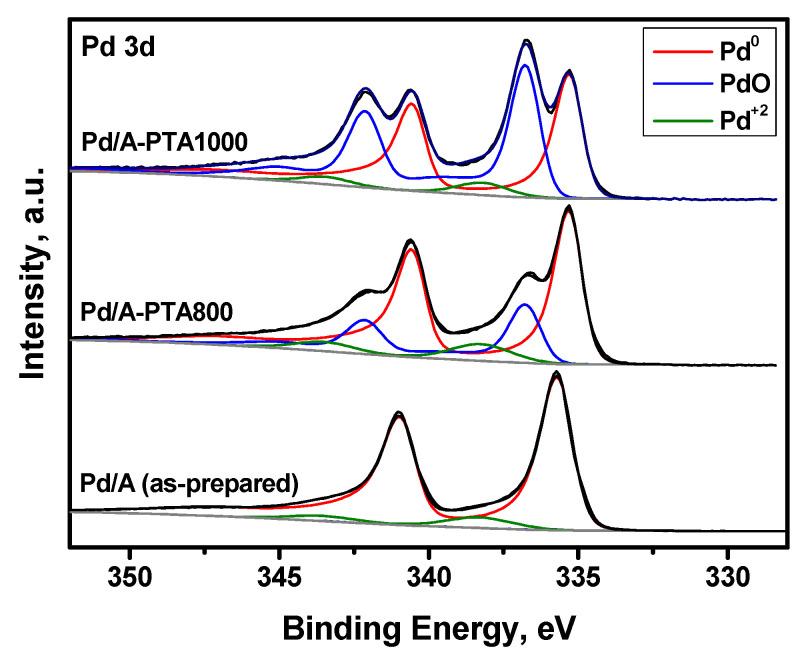
XPS spectra (Pd3d region) of the as-prepared and PTA-aged samples of the Pd/A catalyst.

Then, the as-prepared and PTA-aged samples were studied by high-resolution XPS. The high content of palladium on the surface of the Pd/A catalyst results in the presence of a distinct signal of palladium in the spectra despite such a low Pd loading (Figure 7). Table 4 presents the numeric data. The high [Pd]/[Al] ratio of 4.65 on the catalyst surface confirms the crust-like nature of the coatings formed by the LED method. The deconvolution of the spectra in the Pd3d region demonstrated that the spectrum of the as-prepared sample is close to that of metallic Pd^0^. At the same time, the Pd3d binding energy (335.7 eV) is considerably higher compared to bulk metallic palladium (335.1 eV). In addition, the satellite peak at 346.7 eV, characteristic of metallic palladium, is not observed. These differences between the experimental spectra and the spectra of bulk Pd can be attributed to the small particle size in the studied sample. As is estimated, only 8% of Pd is in the oxidized state in the form of Pd^2+^.

After PTA-aging at 800 °C, the percentage of PdO increased noticeably, but the Pd^2+^ content is almost the same as for the as-prepared sample. At the same time, the [Pd]/[Al] ratio decreased by an order of magnitude. This can be attributed to the sintering of Pd particles and their migration into the bulk or near-surface layer of the Al_2_O_3_ granules. After aging at 1000 °C, further oxidation of Pd occurred. However, the content of Pd^2+^ diminished to 5%. The observed decrease in the content of dispersed Pd species after high-temperature aging is naturally expected. For the Pd/A-PTA800 and Pd/A-PTA1000 samples, the binding energy of Pd^0^ decreased to 335.3 eV, which is closer to the known value for bulk PdO. This can be attributed to the enlargement of Pd particles. On the contrary, as reported recently for bimetallic Pd-Rh particles supported on La_2_O_3_- and ZrO_2_-promoted aluminas [24], all Pd species remained in the metallic state after the PTA procedures and retained an overestimated value of binding energy.

**Figure 8:**
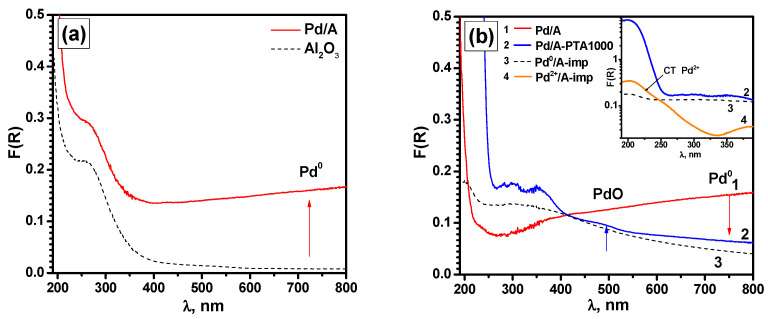
Diffuse reflectance UV-vis spectra: (**a**) of the as-prepared Pd/Al sample and pure Al_2_O_3_ support; (**b**) of the as-prepared Pd/A (1) and aged Pd/A-PTA1000 (2) samples (the spectra are presented after subtraction of the spectrum for pure Al_2_O_3_ support). The spectra for the reference samples Pd^0^/A-Imp (3) and Pd^2+^/A-Imp (4) are given for comparison. Inset shows the UV region of the spectra for the Pd/A-PTA1000 (2), Pd^0^/A-Imp (3), and Pd^2+^/A-Imp (4) samples.

Distinct from XPS, the UV-vis spectroscopic technique allows characterizing the average state of the metal in the entire volume of the catalyst, and not just the part of it that is localized on the outer surface of the granules. The diffuse reflectance spectrum of the as-prepared sample (Figure 8a) shows a wide non-structural band in the red region of the spectrum, which is characteristic of agglomerated Pd^0^ particles. As already mentioned, the particle size in the Pd/A catalyst is about 2 nm [74]. At the same time, the particles cover the surface of the granules almost completely, which explains the appearance of a non-structural band in the long-wavelength region. Such a band is usually observed for large Pd^0^ particles only. Figure 8b shows the diffuse reflection UV-vis spectra of the as-prepared Pd/A sample (spectrum #1) and the same sample after aging at 1000 °C (spectrum #2) after the subtraction of the spectrum of pure alumina support. For a better interpretation of the spectra, two reference samples were prepared by the impregnation method. The synthesis procedure is described in more detail in Section 2. The first one, Pd^2+^/A-Imp, contains Pd^2+^ in the form of oxide clusters. In the second one, Pd^0^/A-Imp, palladium is in the form of metal Pd^0^ particles in a highly dispersed state. The presented spectra of the reference samples were obtained by subtracting the spectrum of pure Al_2_O_3_ and corrected for a Pd concentration of 0.03 wt.% by reducing the intensity.

In the case of the as-prepared Pd/A sample (Figure 8b, spectrum #1), palladium is in the form of closely located particles Pd^0^. Contrarily, after aging at 1000 °C (spectrum #2), partial oxidation of palladium occurs. This is accompanied by a decrease in the absorption intensity of the non-structural band in the red region of the spectrum and the formation of a band of small intensity in the d–d transition region for PdO at 480 nm. Along with this, the remaining region of the visible absorption spectrum is very close to that of the reference sample Pd^0^/A-Imp (spectrum #3). The intense UV absorption band of Pd/A-PTA1000 (Figure 8b, inset) is most likely associated with organic contaminants and does not correspond to either the charge transfer band characteristic of Pd^2+^ complexes (spectrum #4) or the absorption of metal particles of Pd^0^ in a highly dispersed state (spectrum #3).

**Figure 9:**
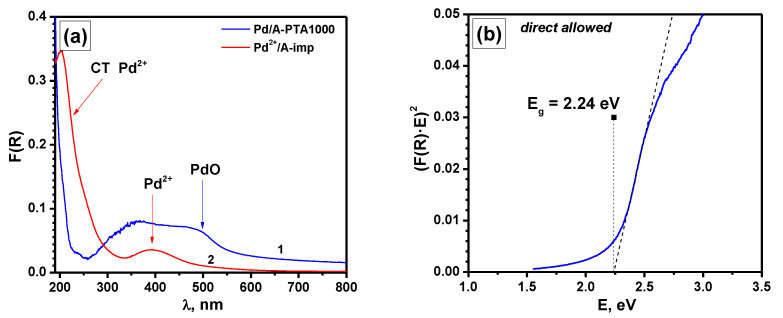
(**a**) Diffuse reflectance UV-vis spectra of the Pd/A-PTA1000 sample after calcination in air at 500 °C (1) and the reference sample Pd^2+^/A-Imp (2). Note that the spectra are presented after subtraction of the spectrum for pure Al_2_O_3_ support. (**b**) Dependence of (F(R)∙E)^2^ on photon energy E, characterizing the value of band gap *E_g_* for the Pd/A-PTA1000 sample.

In order to oxidize the supported palladium and remove organic contaminants, the Pd/A-PTA1000 sample was additionally calcined in air for 4 h at 500 °C. This procedure makes it possible to completely oxidize Pd^0^ particles of small sizes without sintering the formed PdO particles and preserving their size. The spectrum of this sample is shown in Figure 9a. As is seen, a wide band in the region of d–d transitions with a boundary in the region of 500 nm was formed. Moreover, the spectrum is characterized by an almost complete absence of a charge transfer band characteristic of Pd^2+^ complexes. Such a feature can correspond to PdO particles with characteristic *E_g_* values of ~2.24 eV (Figure 9b) in the complete absence of Pd^2+^ species in the form of oxide clusters and isolated ions.

**Figure 10:**
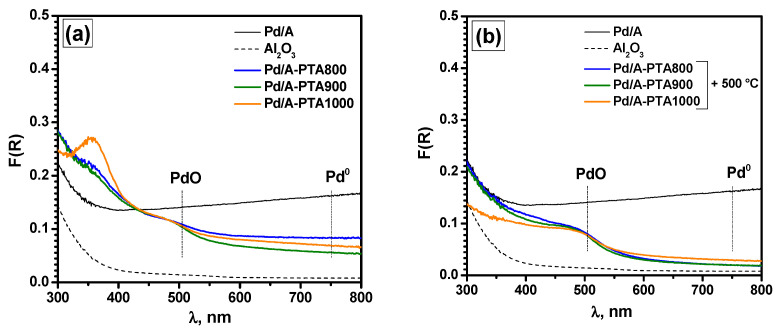
Diffuse reflectance UV-vis spectra of the as-prepared Pd/A sample, pure Al_2_O_3_ support, the Pd/A sample after PTA (**a**) and after PTA with subsequent calcination at 500 °C (**b**).

At the final step of the research, all three samples after PTA-aging at different temperatures, Pd/A-PTA800, Pd/A-PTA900, and Pd/A-PTA1000, were ground in a mortar and studied by UV-vis spectroscopy. Then, they calcined in the air for 4 h at 500 °C, and UV-vis spectra were recorded again. As in the previous case, the UV absorption band is presumably related to the presence of organic contaminants (e.g., adsorbed toluene, carbonyl or carboxyl groups) and does not correspond to either the charge transfer band characteristic of Pd^2+^ complexes or the Pd^0^ absorption. As is seen from Figure 10a, after PTA-aging, a band corresponding to PdO is formed with an edge in the region of 500 nm. Along with this, a decrease in the absorption intensity in the region of 750 nm characteristic of the Pd^0^ is observed (Table 5). In this case, the minimum corresponds to the PTA temperature of 900 °C. The further temperature rise slightly increases the proportion of agglomerated Pd^0^. The absorption value for the reference sample Pd^2+^/A-Imp (Figure 9a, spectrum #2) in the complete absence of Pd^0^ is F(R)~2.0×10^−3^. After calcination at 500 °C (Figure 10b), when all Pd^0^ particles of small size are oxidized completely, the absorption intensity in the region of 750 nm drops significantly. At the same time, we observe a slight increase in intensity for the Pd/A-PTA1000 sample (Table 5). This allows us to conclude that during the PTA-aging at 1000 °C, a small proportion of large agglomerates/particles Pd^0^ is formed, most likely with dimensions greater than 100 nm, which are no longer oxidized in the conditions under consideration. This is qualitatively confirmed by the dependence of the value of the band gap (*E_g_*) for PdO particles (Table 4), although the changes are quite small here. This phenomenon is in good agreement with the results of the TEM study, which also indicate the formation of very large Pd^0^ particles after aging at 1000 °C. The UV-vis data are also consistent with the XPS data, which indicate the oxidation of palladium as a result of thermal aging. However, the differences in UV-vis spectra for the Pd/A-PTA800, Pd/A-PTA900, and Pd/A-PTA1000 samples are quite small, which generally correlates with the results of catalytic tests. It can be assumed that part of the palladium migrates into the volume of the granule and becomes inaccessible for studies by the XPS method, but can be registered by the UV-vis technique.

## 4. Conclusions

In this work, the laser electrodispersion method was used to synthesize a Pd/Al_2_O_3_ catalyst containing an ultra-low amount of palladium (0.03 wt.%). This method allowed the surface of the alumina granules to be uniformly coated with palladium nanoparticles. The “crust” type of the catalyst was confirmed using TEM and XPS methods. According to these methods, as well as the UV-vis spectroscopy method, the active component is represented by metal particles, and only a small part of palladium is in an oxidized state. The samples were subjected to catalytic tests in both model and real mixtures under prompt thermal aging conditions. In the case of the hydrocarbon-free model mixture, changes in the shape of the heating/cooling CO conversion curves were observed during the catalytic cycles, including a change in the type of temperature hysteresis from direct to inverse. At the same time, in the case of the real mixture, direct hysteresis was detected in all cases. However, in both cases, the catalyst showed high thermal stability.

It should be noted that despite the high-temperature reaction conditions, the alumina support maintained sufficiently high values of specific surface area. Thus, after aging at 1000 °C, the SSA value was as high as 145 m^2^/g. The results of the study by TEM, XPS, and UV-vis spectroscopy methods showed that during high-temperature aging, palladium particles sinter to form oxide and metal particles of several tens of nanometers in size. Herewith, an increase in the aging temperature leads to the sequential enlargement of Pd particles and their oxidation to form PdO. At the same time, the migration of palladium into the bulk of the support’s granules or the near-surface layers cannot be precluded. Examination of the samples in the testing reaction of ethane hydrogenolysis has shown that the available metal surface is maintained during high-temperature catalytic runs under the real mixture conditions, which ensures the high stability of the catalyst system. Summing up the results of the research, it should be once again noted that the synthesized catalyst at significantly lower palladium content shows high catalytic activity and thermal stability, superior to palladium-containing catalysts prepared by conventional chemical methods. The obtained results open new horizons for the design of a new generation of catalysts for the treatment of vehicle exhaust gases. Such catalysts will be cheaper and more environmentally friendly than those currently used. As for the possibility of scaling the suggested technology to the industrial level, the estimates show that one LED unit can provide annual production of TWCs of the described composition in the amount sufficient to equip at least 50,000 vehicles.

## Figures and Tables

**Table 1:** Positions of the maxima on the differential light-off curves for CO oxidation in the PTA mode over the Pd/A catalyst (the most intensive maximum is highlighted by bold letters).

Run Number	Reaction Conditions			
	Model Mixture		Real Mixture	
	Heating	Cooling	Heating	Cooling
1	216	212	259	240
2	235	220	258	240
3	240	**218**, 247	261	248
4	231	**219**, 257	268	252
5	236	**232**, 274	268	253
6	255	**232**, 275	278	258
7	255	227, **251**	280	257
8	249	228, **257**	276	260
9	257	225, **245**	286	258
10	258	225, **247**	283	259
11	263	282	282	258

**Table 2:** Comparison of the T_50_ values for the Pd/A sample studied in the present work and previously reported Pd/Al_2_O_3_ catalysts. Note that all the experiments were performed in the PTA mode using the real mixture.

Sample	Run Number						Ref.
	1	3	5	7	9	11	
0.028 wt.% Pd/Al_2_O_3_	257	259	270	277	283	278	This work
0.21 wt.% Pd/Al_2_O_3_	160	238	258	268	269	278	[74]
0.21 wt.% Pd/Al_2_O_3_ + La_2_O_3_ *	164	200	231	232	-	-	[90]
0.3 wt.% Pd/Al_2_O_3_ *	229	265	278	278	-	-	[91]
0.24 wt.% Pd/δ-Al_2_O_3_ *	-	260	269	274	-	-	[56]
0.12 wt.% Pd/Al_2_O_3_ *	243	261	272	274	-	-	[15]

* The sample was tested in seven runs only.

**Table 3:** Textural characteristics of pure Al_2_O_3_ and Pd/A catalyst before and after catalytic tests in the PTA mode.

Sample	SSA, m^2^/g	V_total_, cm^3^/g	D_av_, nm	Ref.
Al_2_O_3_	167	0.53	11	[74]
Pd/A (as-prepared)	191	0.55	11	[74]
Pd/A-PTA800	187	0.56	12	This work
Pd/A-PTA900	173	0.55	12	This work
Pd/A-PTA1000	145	0.49	13	This work

**Table 4:** The values of binding energy (BE) and the portion of palladium in different oxidation states.

Sample	[Pd]/[Al]	BE (Pd3d_5/2_), eV			ν (Pd), %		
		Pd^0^	PdO	Pd^2+^	Pd^0^	PdO	Pd^2+^
Pd/A (as-prepared)	4.65	335.7	336.9	338.3	92	0	8
Pd/A-PTA800	0.45	335.3	336.8	338.2	66	25	9
Pd/A-PTA1000	0.11	335.3	336.8	338.2	47	48	5

**Table 5:** Dependence of the intensity F(R) in the absorption area of Pd^0^ at 750 nm and the value of *E_g_* on the temperature of the PTA procedure.

Sample	F(R) at 750 nm		***E_g_*** **, eV**
	PTA	PTA + 500 °C	
Pd/A-PTA800	0.103	0.020	2.25
Pd/A-PTA900	0.056	0.020	2.28
Pd/A-PTA1000	0.069	0.029	2.24

## Data Availability

Data are contained within the article.

## Supplementary Materials

The following supporting information can be downloaded at: https://www.mdpi.com/article/10.3390/ma16093501/s1, Figure S1: TEM images of the as-prepared Pd/A catalyst; Table S1: Catalyst surface composition (at.%) according to the EDX data for the as-prepared Pd/A sample; Figure S2: Heating and cooling light-off curves for the Pd/A sample tested in a model mixture under the PTA conditions; Figure S3: Heating and cooling light-off curves for the Pd/A sample tested in a real mixture under the PTA conditions; Figure S4: Differential heating and cooling light-off curves for the Pd/A sample tested in a model mixture under the PTA conditions; Figure S5: Differential heating and cooling light-off curves for the Pd/A sample tested in a real mixture under the PTA conditions.
