# Supplementary material for: Studies on High-Temperature Evolution of Low-Loaded Pd Three-Way Catalysts Prepared by Laser Electrodispersion
Source: Materials (Basel). 2023 May 1;16(9):3501. doi: 10.3390/ma16093501 (PMC10179799; doi:10.3390/ma16093501)
Supplement: Supplementary file 1 [file materials-16-03501-s001.zip › materials-2339840-supplementary.pdf]

materials

MDPI

Supplementary Information

Studies on High-Temperature Evolution of Low-Loaded Pd Three-Way Catalysts Prepared by Laser Electrodispersion

Tatiana N. Rostovshchikova [1,*], Marina I. Shilina [1], Sergey A. Gurevich [2], Denis A. Yavsin [2], Grigory B. Veselov [3], Vladimir O. Stoyanovskii [3], Aleksey A. Vedyagin [3]

[1] Chemical Department, Lomonosov Moscow State University, 1/3 Leninskie Gory, 119991 Moscow, Russia
[2] Ioffe Physico-Technical Institute, Russian Academy of Sciences, 26 Politechnicheskaya Str., 194021 Saint Petersburg, Russia
[3] Boreskov Institute of Catalysis, 5 Lavrentyev Ave., 630090 Novosibirsk, Russia
* Correspondence: t.rost50@mail.ru

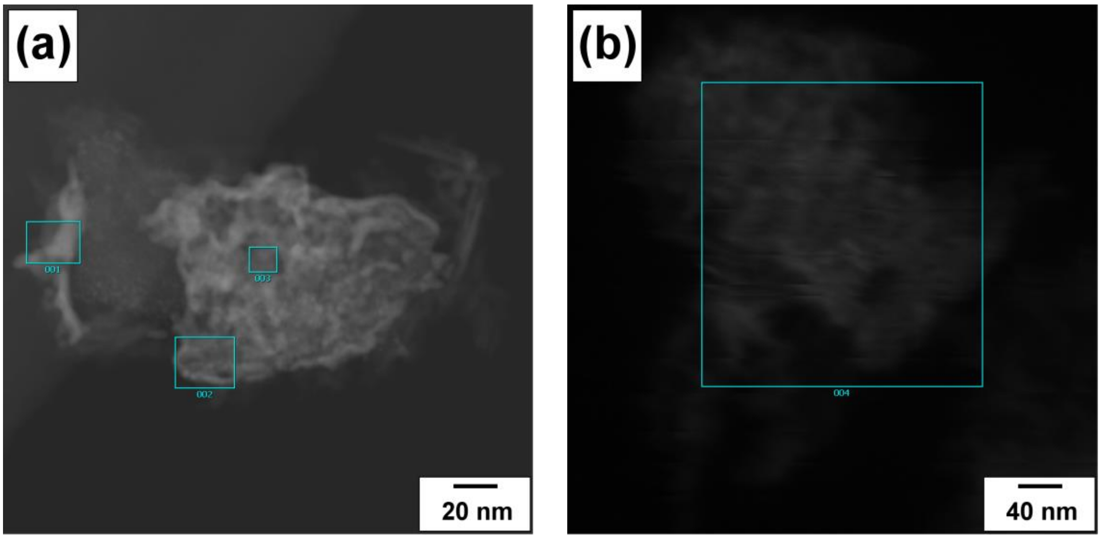

Figure S1. TEM images of the as-prepared Pd/A catalyst. The EDX data for the highlighted areas are presented in Table S1.

Table S1. Catalyst surface composition (at.%) according to the EDX data for the as-prepared Pd/A sample.

Area	Element, atom.%				Pd/Al
	C	O	Al	Pd	
1	82.30	10.39	2.67	4.64	1.7
2	22.07	35.29	20.46	22.17	1.1
3	19.53	49.07	15.91	15.49	1.0
4	64.72	22.56	11.61	1.11	0.1

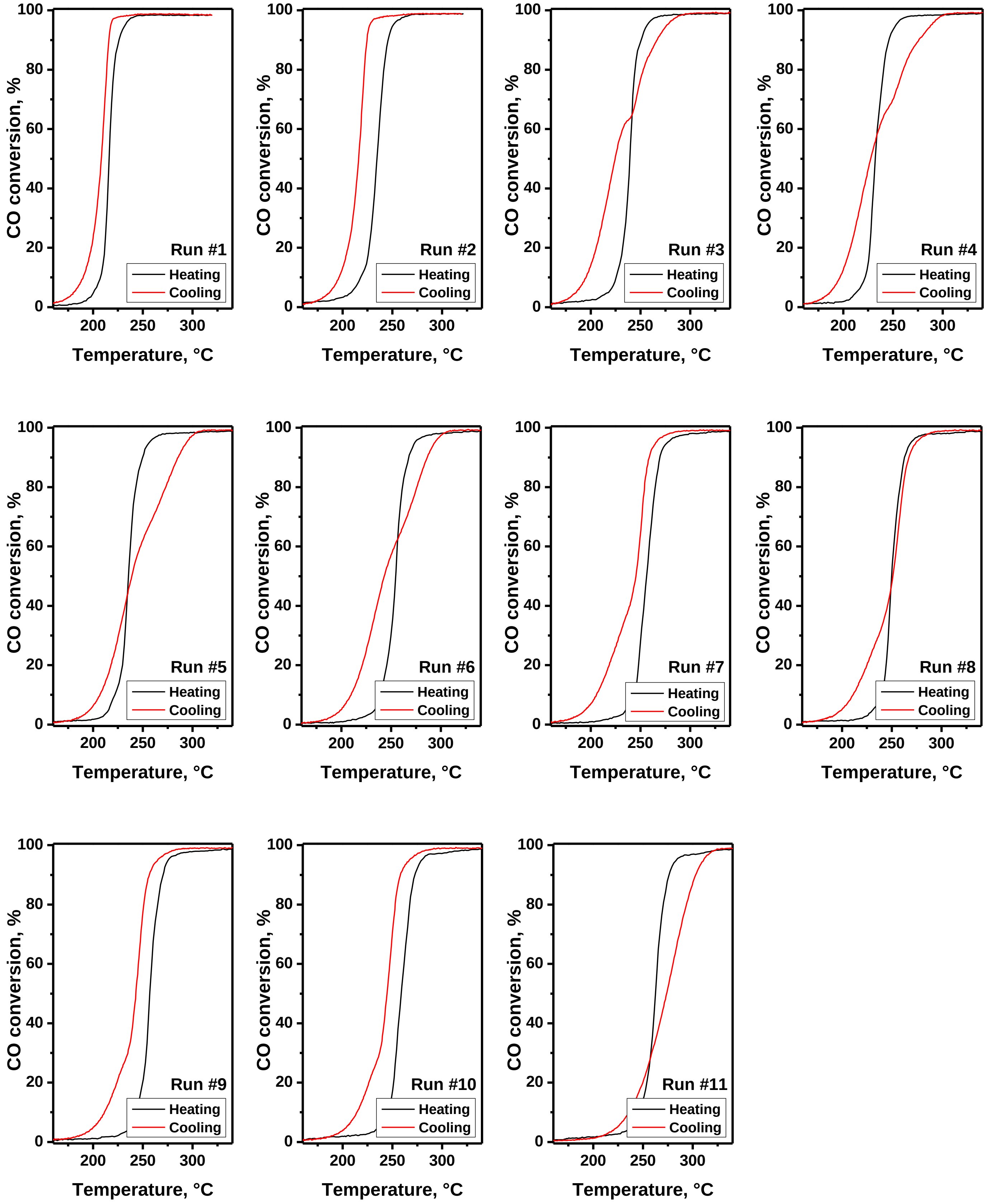

Figure S2. Heating and cooling light-off curves for the Pd/A sample tested in a model mixture under the PTA conditions.

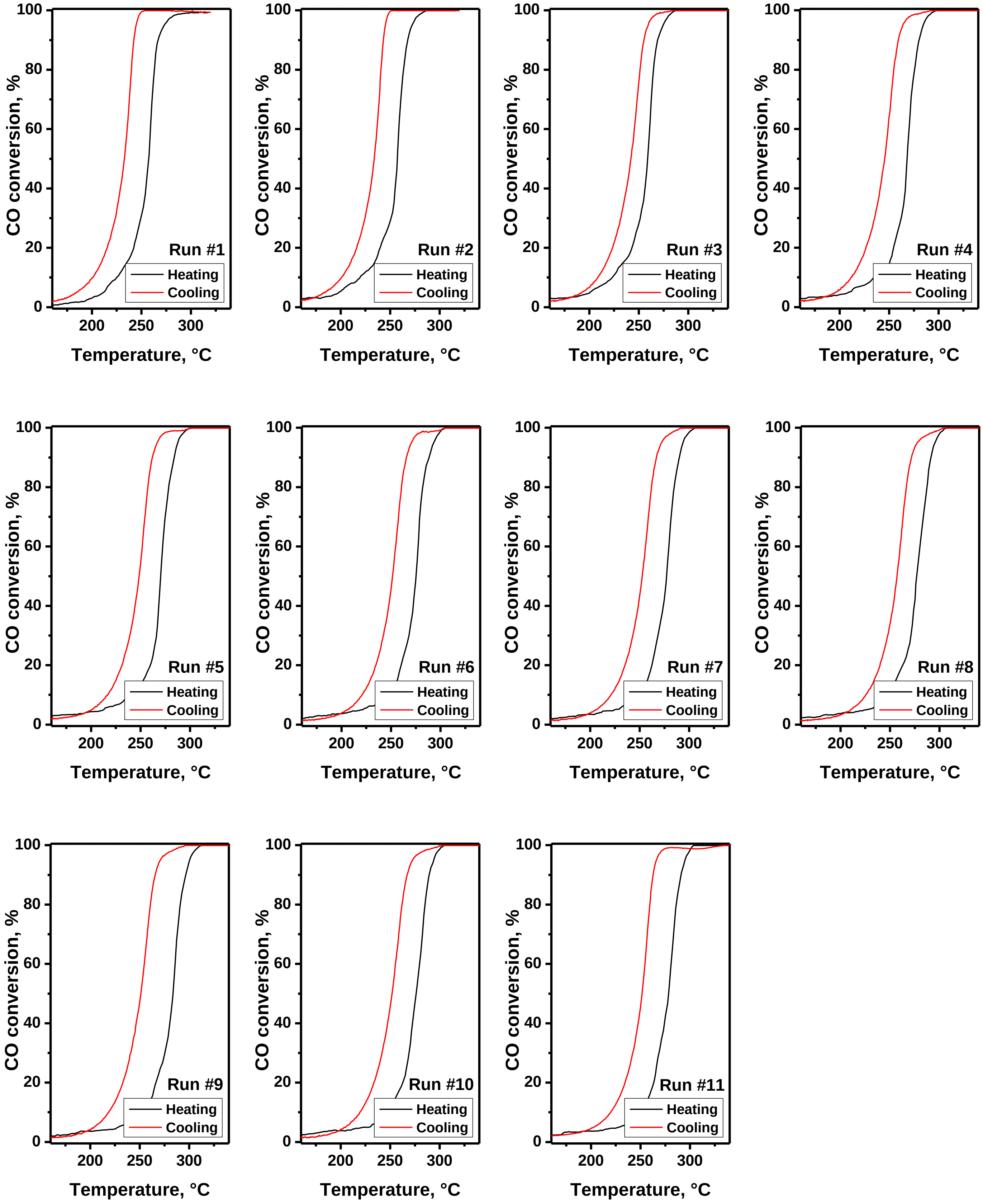

Figure S3. Heating and cooling light-off curves for the Pd/A sample tested in a real mixture under the PTA conditions.

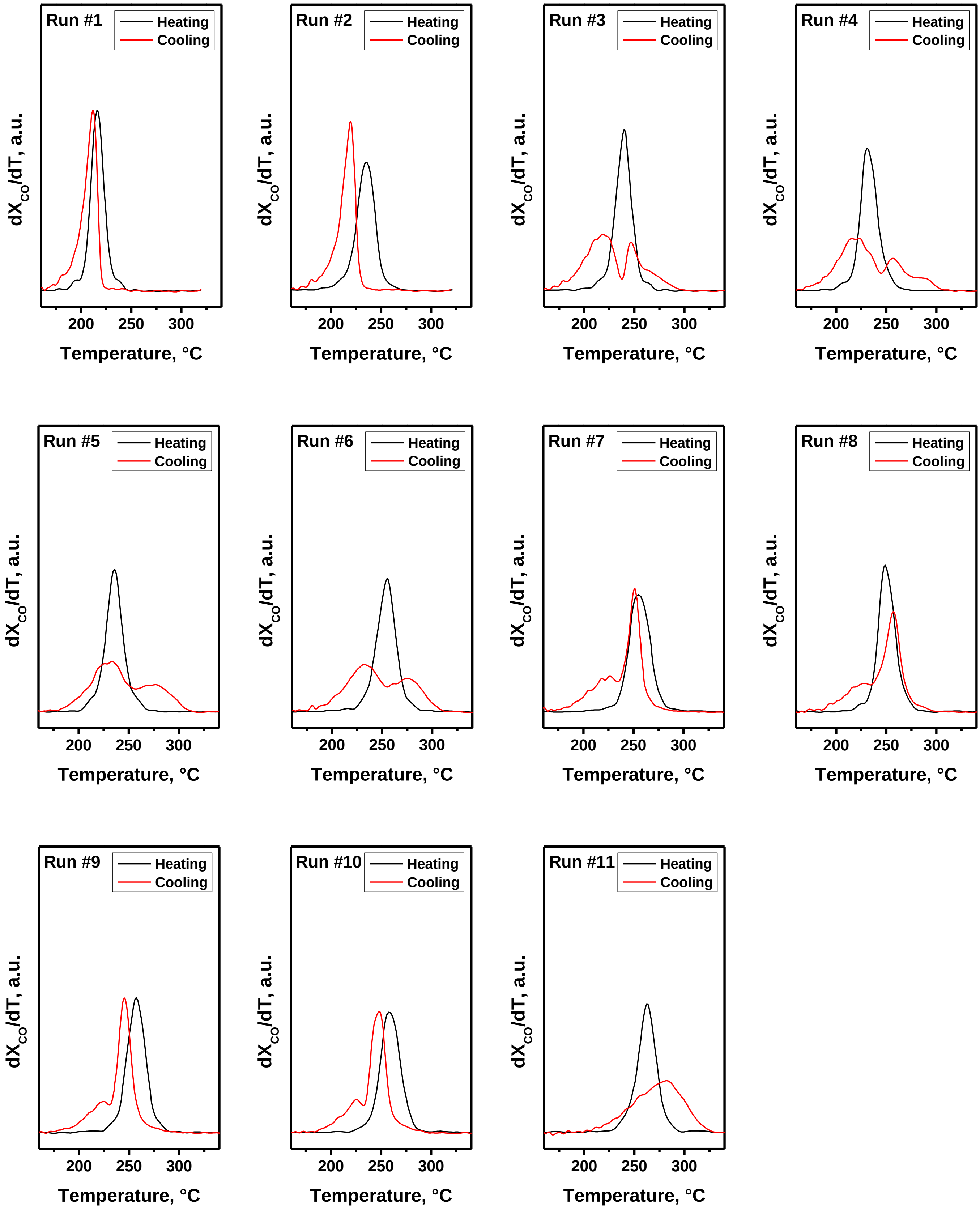

Figure S4. Differential heating and cooling light-off curves for the Pd/A sample tested in a model mixture under the PTA conditions.

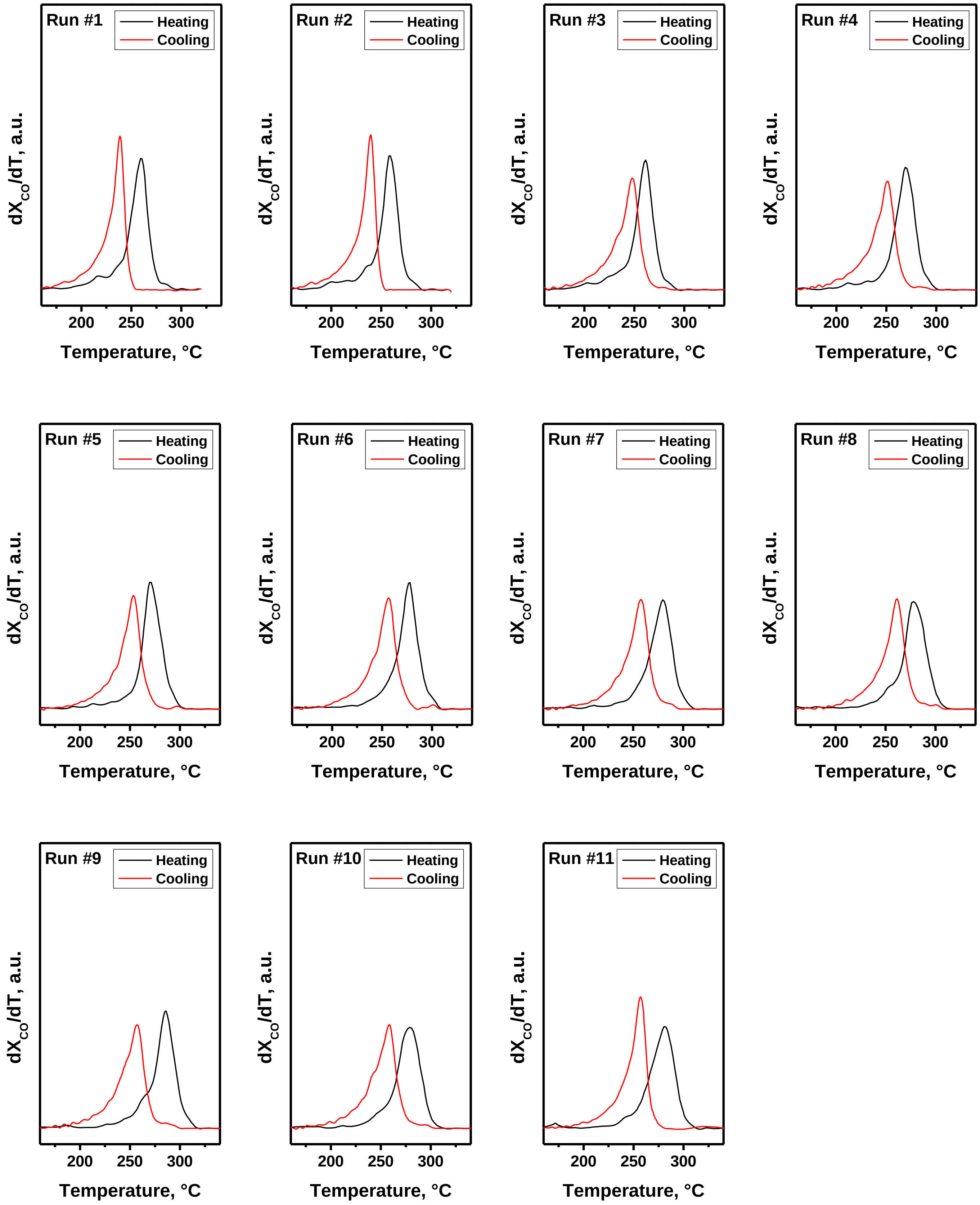

Figure S5. Differential heating and cooling light-off curves for the Pd/A sample tested in a real mixture under the PTA conditions.
